# Red cell allo- and autoimmunisation in transfused sickle cell and cancer patients in Kenyatta National Hospital, Nairobi, Kenya

**DOI:** 10.4102/ajlm.v4i1.297

**Published:** 2015-09-25

**Authors:** Caroline Mangare, Amos Mbugua, Peter Maturi, Jamila Rajab, Rainer Blasczyk, Hans-Gert Heuft

**Affiliations:** 1 Jomo Kenyatta University of Agriculture and Technology, Nairobi, Kenya; 2 Kenyatta National Hospital/University of Nairobi, Department of Hematology and Blood Transfusion, Nairobi, Kenya; 3 Institute for Transfusion Medicine, Hannover Medical School, Hannover, Germany

## Abstract

**Background:**

Currently, no data are available on the prevalence of red blood cell (RBC) antibody formation amongst Kenyan patients with multiple transfusion needs, such as patients with sickle cell disease (SCD) or haematological malignancies (HM) and solid (SM) malignancies.

**Objectives:**

We determined the prevalence and specificities of RBC alloantibodies and autoantibodies in two patient groups with recurrent transfusion demands at Kenyatta National Hospital, Nairobi, Kenya.

**Method:**

Between February and August 2014, 300 samples from SCD, HM and SM patients were collected and screened for alloantibodies. Samples from 51 healthy blood donors were screened for irregular antibodies and phenotyped.

**Results:**

Amongst the 228 patients with viable samples (SCD, *n* = 137; HM, *n* = 48; SM, *n* = 43), the median transfusion frequency was two to three events per group, 38 (16.7%) were RBC immunised and 32 (14.0%) had a positive direct antiglobulin test. We identified specific alloantibodies in six patients (2.6%). Four of these six were SCD patients (2.9%) who had specific RBC alloantibodies (anti-C^w^, anti-M, anti-Co^b^, anti-S); amongst HM patients one had anti-K and one had anti-Le^a^. RBC autoantibody prevalence was 3.1% (7/228). Amongst the healthy blood donors, the R_o_r, ccD.ee and R_2_r, ccD.Ee phenotypes accounted for 82% of the Rhesus phenotypes and all were Kell negative.

**Conclusion:**

The numbers of transfusions and the rates of RBC alloantibodies are low and the most important RBC alloantibody-inducing blood group antigens are relatively homogeneously distributed in this population. A general change in the Kenyatta National Hospital pre-transfusion test regimen is thus not necessary. The current transfusion practice should be reconsidered if transfusion frequencies increase in the future.

## Introduction

Blood transfusion constitutes an important supportive modality in the management of patients with sickle cell disease (SCD) and cancer, because of longer periods of treatment and increased survival rates. SCD is the most prevalent haematologic genetic disease in Kenya^1^ and cancer is an increasingly important challenge for the Kenyan public health system.^2^ Red blood cell (RBC) allo- and autoimmunisation often develop as a result of transfusions with allogeneic blood and occur because of the response of recipients’ immune systems to foreign RBC antigens from donors.^3^ Some of the facets involved in these immunological reactions are: recipient age; sex; history of pregnancy; number of blood units transfused; and diagnosis- and treatment-related impairment to the recipient's immune system.^4,5^

RBC alloimmunisation is associated with clinical complications, such as morbidity resulting from acute and delayed haemolytic transfusion reactions. The former can mimic a sickle cell crisis. Furthermore, alloimmunisation creates difficulties for laboratories, including expensive and time-consuming laboratory workups to determine compatible blood, especially for cases with multiple alloantibodies (alloAb). AlloAbs can become undetectable over time and/or be boostered as an anamnestic response after another transfusion. RBC alloAb against incompatible RBC in an allogeneic bone marrow transplant may require procedures for RBC reduction.^6,7^ The development of alloAb has been associated with that of autoantibodies (autoAb),^8,9^ which can shorten the lifespan of recipients’ own RBCs and/or transfused RBCs and potentially cause haemolysis. Because of this, these patients may require several transfusions and may need interventions, such as drugs to suppress the immune system and/or splenectomy.^9^ These challenges need to be considered when handling patients who are likely to be transfusion-dependent, as well as those who could benefit from haematopoietic stem cell transplantations. Decreasing the risk of RBC alloimmunisation by implementing strategies to avoid allogeneic blood transfusions (e.g., erythropoietin administration in cancer patients) or extensive phenotypic matching of RBC blood group antigens, such as the Rhesus, Kell, Duffy, Kidd and MNS blood groups, has been advocated previously.^10,11^ However, this is costly and impractical in many health settings, particularly in developing countries.

Studies conducted on the frequency of RBC alloimmunisation in different patient populations have reported rates of 1% to 6% in occasionally transfused patients and up to 30% in poly-transfused patients.^12^ In Europe and the United States, alloimmunisation rates of 5% to 36% have been reported amongst transfused SCD patients.^13^ Currently, there are minimal data from Africa regarding transfusion-dependent RBC allo-/autoimmunisation. The few existing studies have reported varied results. A Ugandan study^3^ recently reported an RBC alloimmunisation prevalence rate of 6.1% amongst 428 SCD patients. An investigation conducted in Egypt amongst 42 SCD patients reported an alloimmunisation rate of 21.4%,^14^ whereas amongst 130 Tunisian thalassaemia patients, RBC alloimmunisation was 7.7% and 40% of these patients developed RBC autoantibodies.^15^ In a study of 108 Ugandan patients with malignancies, alloimmunisation was reported at a frequency of 8.3%.^16^ There are no data on the prevalence of RBC alloAb/autoAb formation amongst Kenyan patients, where pretransfusion testing is limited to ABO/Rhesus D group typing and crossmatching only. As there is no routine pre-transfusion RBC antibody screening or identification, this study sought to determine the prevalence and specificities of RBC alloAbs and autoAbs amongst two different groups of transfusion recipients at Kenyatta National Hospital (KNH), Nairobi, Kenya. In addition, we screened samples from blood donors for irregular antibodies and phenotyped them for ABO, Rhesus and Kell antigens in order to determine whether there is alloimmunisation in the general population served by KNH.

## Methods

### Setting and design

Using a cross-sectional design, SCD, haematological malignancy (HM) and solid malignancy (SM) patients attending haematology and oncology clinics at KNH were approached between February and August 2014 and invited to participate in the study. To be eligible for the study, participants had to be KNH patients with SCD, HM or SM who had received at least one allogeneic blood transfusion; 300 patients met the inclusion criteria. Samples from 51 healthy blood donors of African ancestry from KNH's blood bank were obtained for limited RBC antigen phenotyping. The Kenya National Blood Transfusion Policy defines the criteria for healthy donors as those who are aged 18–65 years; weigh more than 50 kg; have a minimum haemoglobin of 12 g/dL; have normal blood pressure (systolic 120–129 mmHg, diastolic 80–89 mmHg) and a pulse rate of 60–100 beats per minute.^17^

### Data and sample collection

After obtaining informed consent, 2–4 mL of blood was drawn from patients into ethylenediaminetetraacetic acid tubes for laboratory investigations. Patients’ notes were reviewed for: demographic characteristics; recipient age; sex; diagnosis; history of pregnancy; and transfusion history and indications. The number of blood components, units transfused and transfusion episodes were recorded. Healthy blood donor samples were collected from donors who gave consent and met the healthy donor criteria. Documentation of patient ethnicity is a routine requirement in Kenyan medical records or clinical data.

### Laboratory investigations

Plasma and RBCs were separated within two hours after collection and the plasma was frozen whilst the red cells were stored at 2 °C – 6 °C. RBCs were preserved by adding a drop of citrate phosphate dextrose anticoagulant. The samples were then shipped on dry ice at a controlled temperature to the Institute for Transfusion Medicine at Hannover Medical School, Hannover, Germany for immunohaematological analysis.

### Immunohaematological testing

The Bio-Rad ID gel card system (DiaMed-ID^®^; Bio-Rad Laboratories, DiaMed GmbH, Cressier, Switzerland) was used with both untreated and papain-treated RBC reagents. Plasma samples were screened for the presence of RBC alloAb by use of a standard three-cell panel of reagent group O RBCs using NaCl gel cards at room temperature for alloAbs with low thermal range and low ionic strength saline (LISS) gel cards (LISS/Coombs) at 37 °C for warm-reacting alloAbs. For samples that showed agglutination, subsequent antibody identification was carried out with at least one 11-cell group O RBC panel (usually Bio-Rad, Switzerland). In instances without immediate determination of alloAb specificity, additional cell panels (e.g., an 11-cell [Grifols Inc., Los Angeles, California, United States] and/or a 16-cell panel [Sanquin, Plesmanlaan, Amsterdam, Netherlands]) were used. If a patient's plasma sample showed agglutination of reagent screening cells, an autocontrol was also performed by reacting the patient's RBCs with his or her own plasma. Positive autocontrols were further evaluated by means of a poly-/monospecific direct antiglobulin test (DAT). DAT was performed using monoclonal gel cards consisting of anti-IgG, anti-IgA, anti-IgM, anti-C3c and anti-C3d. From samples that were positive with at least one of these antiglobulins, an acid eluate was prepared. The eluate was screened using the standard three-cell panel. Those that were positive were then analysed for specificity using 11-cell panels containing these antigens: D, C, E, c, e, K, k, Fy^a^, Fy^b^, Jk^a^, Jk^b^, Le^a^, Le^b^, P1, M, N, S and s. In addition, donor blood was screened for irregular antibodies using a panel of three screening cells and then phenotyped for ABO, Rhesus (C, c, D, E, e) and Kell antigens. Plasma samples of 40 blood donors were screened for RBC alloAb.

Patients were considered to be alloimmunised if antibodies to one or more RBC antigens could be identified, whilst autocontrol and DAT screening remained negative. Patients with a positive autocontrol, a positive DAT and a reactive acid eluate were considered to be sensitised to have autoAbs to RBCs. In cases with a positive autocontrol and a positive DAT, but a non-reactive acid eluate, a non-specific loading of the RBC surface with immunoglobulins was assumed.

‘Immune’ antibodies are formed after immunisation through pregnancy and/or previous transfusions. ‘Naturally-occurring’ antibodies are formed as a result of exposure to environmental agents similar to red cell antigens, such as bacteria.

### Statistical analyses

Statistical software packages were used: Excel 5.0 (Microsoft, Redmond, California, United States 1993) for data management and Statistical Package for the Social Sciences 12.0 (SPSS Inc., Chicago, Illinois, United States 2003) was used for analysis. Student's *t*-test was used for variables with normal distribution. Categorical variables of possible associations between RBC alloimmunisation and sex, units of blood transfusion, diagnosis of SCD or solid and haematological malignancy were compared using the Chi-squared test. Groups were assumed to differ significantly when the probability level was less than 0.05.

### Ethical considerations

Ethical approval was obtained from KNH/University of Nairobi Ethics Review Committee. Both oral and written informed consent was obtained from patients or their guardians. Donors completed a questionnaire provided by the blood bank services and signed a consent form.

## Results

### Patient data

**FIGURE 1:**
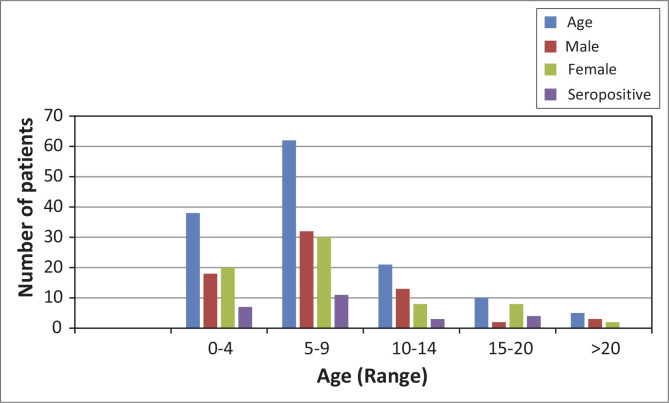
Transfused sickle cell disease patients by age, sex and seropositivity at Kenyatta National Hospital, Nairobi, Kenya, 2014.

**FIGURE 2:**
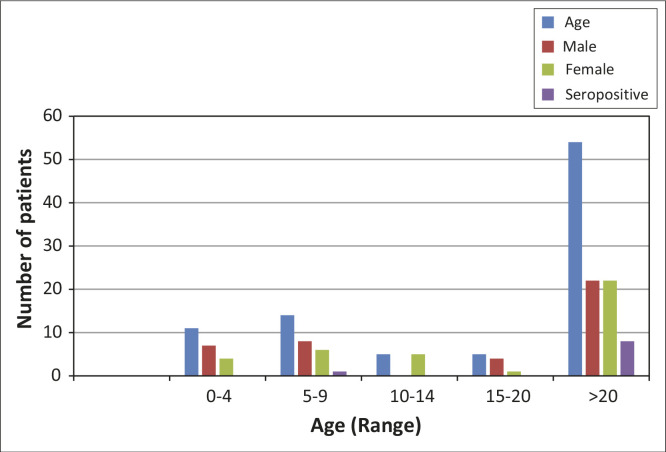
Transfused cancer patients by age, sex and seropositivity at Kenyatta National Hospital, Nairobi, Kenya, 2014.

Of the samples from 300 patients who met the inclusion criteria, 72 samples could not be evaluated for the following reasons: insufficient sample because of leakage during shipment (*n* = 40); samples breaking in the centrifuge whilst processing (*n* = 20); and lack of proper labeling (*n* = 12). A total of 228 samples were analysed, including 137 from SCD patients, 48 from HM patients and 43 from SM patients, with a median number of two to three transfusions per group (Table 1). Of these, 117 (51.3%) were women, of whom 22 (18.8%) had a history of pregnancy. Overall, the mean age at the time of blood transfusion was aged 17.2 years (range: 1–93). Cancer patients, in particular SM patients, were significantly older than SCD and HM patients (*P* < 0.001). Indeed, the majority of patients were children aged 16 years or younger (*n* = 159; 70%); 14% were aged three years or younger (Figures 1 and 2). There were no significant differences in the female to male ratios between the groups.

**TABLE 1:** Characteristics of transfused sickle cell disease and cancer patients at Kenyatta National Hospital, Nairobi, Kenya, 2014.

Variables	All patients	SCD patients	Cancer patients		
			All cancer patients	HM patients	SM patients
**Characteristics**					
Number	228	137	91	48	43
Mean Age (range)	17.2 (1-93)	8 (1-36)	31.1 (1.5-93)	22.4 (1.5-70)	48.1 (8-93)
Female / male ratio	1.05	1.01	1.17	1.28	1.15
**Transfusions**					
Number	685	331	354	227	127
Mean (range)	3 (1-19)	2.4 (1-8)	3.9 (1-19)	4.7 (1-19)	3.0 (1-10)
Whole blood	532	308	224	147	77
Packed red blood cells	85	23	62	37	25
Platelets	68	0	68	43	25
Immunisation	-	20.4%	11.0%	-	-

SCD, Sickle cell disease; HM, Haematological malignancy; SM, Solid malignancy.

Patients received ABO⁄Rhesus D compatible and non-leucocyte-depleted whole blood units (*n* = 532), packed RBC transfusions (*n* = 85) and platelet transfusions (*n* = 68), totaling 685 units of blood in 593 transfusion events (i.e., 1 transfusion unit per transfusion episode or a mean of 2.7 transfusion units per patient). Of the SCD patients, 90% were transfused because of severe anaemia – haemoglobin less than 5–6 g/dL according to World Health Organization guidelines.^18^ Transfusion in malignancy patients was mainly a result of anaemia caused by intensive chemotherapy, by the disease process and/or surgical interventions. All cancer patients were receiving chemotherapy at the time of enrollment into the study. HM patients had the following types of malignancies: acute lymphoblastic leukaemia (*n* = 15), chronic lymphoblastic leukaemia (*n* = 10), Hodgkin's lymphoma (*n* = 9), multiple myeloma (*n* = 6), acute myeloid leukaemia (*n* = 5) and chronic myeloid leukaemia (*n* = 3). SM patients had the following types of malignancies: abdominal tumour (*n* = 1), bladder (*n* = 2), breast (*n* = 8), cervical (*n* = 4), colon (*n* = 3), hepatocellular (*n* = 2), mouth (*n* = 1), nasopharyngeal (*n* = 1), pancreatic (*n* = 2), prostate (*n* = 4), rectum (*n* = 4), rhabdomyosarcoma (*n* = 4), sinonasal tumour (*n* = 1), squamous cell carcinoma (*n* = 2) and stomach cancer (*n* = 4). The *P*-value for the number of blood units transfused was 0.004, which was statistically significant as cancer patients received more transfusions.

### Serological results

The overall prevalence of RBC immunisation was 16.7%, with 38 of the 228 patients testing positive for antibody screening. The prevalence of RBC immunisation amongst SCD patients was 20.4% (28 of 137 patients) and amongst malignancy patients, 11.0% (DAT+, *n* = 8 plus alloAb+, *n* = 2; altogether 10 out of 91 patients).

### RBC alloantibody identification

Only 38 patients were positive for the antibody screening, and RBC alloantibodies were detected in only 6 of 228 patients (2.6%) (Table 2). The rate of alloAb formation amongst SCD patients was 2.9% (4 of 137) and 4.2% (2 of 48) amongst HM patients, whereas the prevalence amongst SM patients for alloAb identification was 0. The specificities of the alloAbs from the SCD patients were anti-C^w^, anti-S, anti-Co^b^ (probably immune in nature) and anti-M (probably naturally occurring). In addition, there was one anti-K (immune) and one anti-Le^a^ (natural) in the two HM patients, whereas the SM group showed no RBC alloimmunisation. The rate of alloimmunisation was 6.14% for men versus 8.33% for women; the difference was not statistically significant (*P* = 0.25).

**TABLE 2:** Serological results of transfused sickle cell disease and cancer patients at Kenyatta National Hospital, Nairobi, Kenya, 2014.

Variables	All patients	SCD patients	Cancer patients	
			HM patients	SM patients
Number	228	137	48	43
AlloAb	6	4	2	0
Immune†	-	3‡	1§	0
Naturally occurring¶	-	1††	1‡‡	0
DAT positive	32	24	3	5
IgG+	16	10	3	3
IgG +, C3d/C3c	6	5	0	1
C3c only	7	6	0	1
IgM only	1	1	0	0
IgA only	2	2	0	0

SCD, Sickle cell disease; HM, Haematological malignancy; SM, Solid malignancy; AlloAb, Alloantibody; DAT, Direct antiglobulin test

†, Immune antibodies are formed after immunisation through pregnancy or previous transfusions; ‡, AlloAb specificities: anti-C^w^; anti-S, anti-Co^b^; §, AlloAb specificity: anti-K; ¶, Naturally-occurring antibodies are formed as a result of exposure to environmental agents similar to red cell antigens, such as bacteria; ††, AlloAb specificity: anti-M; ‡‡, AlloAb specificity: anti-Le^a^.

### Red cell autoimmunisation

Of the 228 patients, 32 patients (14.0%) presented a positive DAT (Table 2). Fifty per cent of these patients (16 of 32) were positive for anti-IgG alone, whereas 18.8% (6 of 32) showed reactions to anti-IgG plus anti-C3c or C3d. Out of the subset of 21 IgG-positive patients, the acid eluate was reactive in seven, thereby indicating a true RBC autoAb prevalence of 3.1% for this population of patients (7 of 228) and 33.3% (7 of 32) amongst the DAT-positive patients. RBC autoAb prevalence was 5.1% (7 of 137) amongst SCD patients, whereas there were no RBC autoAbs amongst patients with malignancies. Moreover, we observed a few cases (3 of 32) with isolated IgM or IgA reactivity. The majority (24 of 32) of the DAT-positive reactions with anti-IgM and anti-IgA were observed in the SCD group. Eighteen per cent (24 of 137) of the SCD group were DAT positive compared with 8.8% (8 of 91) in the HM/SM group.

### Comparison of combined RBC allo- and autoimmunisation in sickle cell versus cancer patients

The prevalence of RBC immunisation (demonstration of an immune alloAb and a positive DAT) amongst SCD patients was 19.7% (27 of 137) versus 9.9% (9 of 91). Immune alloAbs were found in 2.2% (3 of 137) of the SCD patients versus 1.1% (1 of 91) of the patients with malignancies. With one exception (polyspecific in eluate, but auto-anti-e in serum), these autoAbs showed polyspecificity only. We also performed a comparison for demographic and transfusion variables between patients with and without serological reactivity (Table 3). We did not find a significant link between patients’ sex, age or number of units of blood transfused and the positivity of the antibody screening.

**TABLE 3:** Characteristics of immunised and non-immunised transfused sickle cell and cancer patients at Kenyatta National Hospital, 2014.

Variables	Immunised	Non-immunised	*P*-value†
**Characteristics**			
All patients (*n*, %)	33 (14.47)	195 (85.53)	-
Age (*n*, range)	17 (1–60)	17.2 (1–93)	0.946
Female to male ratio	1.28	1	0.458
**Number of units transfused**			
Number	93	594	0.496
Mean (range)	2.8 (1–7)	3.05 (1–19)	-

†, *P*-values less than 0.05 were considered to be statistically significant

### Healthy donor phenotypes

Amongst the blood donor samples, there were no serological peculiarities. Fifty-one donors were phenotyped for the Rhesus antigens C/c, D, E/e and for the antigen K (Table 4). Of these, 29 donors (57%) showed the Rh phenotype ccD.ee, the other phenotypes were ccD.Ee (*n* = 13), CcD.ee (*n* = 5), ccddee (*n* = 2) and single cases of Ccddee (*n* = 1) and CcD.De (*n* = 1). None of the 51 donors were Kell positive.

**TABLE 4:** Rhesus and Kell phenotypes amongst 51 healthy Kenyan blood donors at Kenyatta National Hospital, 2014.

Phenotype		*n*	%
Ror	ccD.ee	29	57
R2r	ccD.Ee	13	25
R1r	ccD.ee	5	10
rr	ccddee	2	4
r’r	ccddee	1	2
R1R2	ccD.De	1	2
R2R2	ccD.EE	0	0
K negative	(K-k+)	0	0
**Total**		**51**	**100**

K, Kell.

## Discussion

The risk of alloimmunisation is a concern that needs to be addressed and managed, especially amongst patients requiring multiple blood transfusions, such as those with SCD and malignances. This investigation sought to determine the magnitude of RBC immunisation and to identify antibodies amongst two transfused patient groups. In this study, we detected a significant proportion of patients with some degree of RBC autoimmunisation, as shown by a positive DAT in 14.0% of the patients, seven of whom had true RBC autoantibodies. Eighteen per cent of SCD patients were DAT positive compared with 8.8% of HM/SM patients. In contrast, RBC alloAb formation was low, at only 2.6%. Moreover, the specificities of the demonstrated alloAbs do not occur often in daily laboratory results. Anti-C^w^ and anti-S are comparatively rare Rh- and MNS antibodies, respectively; and anti-Co^b^ is a very rare RBC alloAb specificity of the Colton system. The one example of anti-K that we detected was the only common RBC alloAb specificity. Other common RBC alloAb specificities, such as anti-D, anti-E, anti-c, anti-C, belonging to the Rhesus system, or those of the Kell system (other than anti-K), the Duffy or the Kidd blood group systems, were not found in our study population. In this study, the frequency of alloimmunisation across all patients was determined to be 2.6%; and the rate of alloAb formation was 2.2% amongst patients with malignancies and 2.9% amongst SCD patients.

There may be several reasons for these unexpected findings. Firstly, the total numbers of transfusions and the numbers of transfusion events were low, never exceeding the mean values of three transfusion events per patient. This was particularly true for the SCD patients, who are known to be at high risk for RBC alloAb formation.^3,10,19^ However, SCD patients showed the lowest values for transfused RBC units per patient and transfusion events per patient in our study. The RBC alloimmunisation rate of 2.9% amongst our SCD patients is comparable to a study in a Jamaican cohort,^19^ where the rate was 2.6% amongst 115 transfused SCD patients and 1.6% amongst the total number of 190 patients. However, this rate differs considerably from that reported in a Ugandan study of 428 SCD patients, where the prevalence rate was 6.1%.^3^ Although the mean number of transfusions was three blood units in all of these studies, 21 of the 26 alloimmunised patients in the Ugandan study had received up to 10 blood units. This is marginally higher than the maximum number (*n* = 8) of transfusions observed in our study. Our SCD patients received a mean of 2.4 units of transfusions, HM patients received 4.7 units and SM patients received 3.0 units. Therefore, all groups were exposed to minimal antigenic challenge. Numerous studies have reported that the rate of RBC alloimmunisation increases with the number of transfusions.^3,10,19,20,21,22^ This could explain the low alloimmunisation rate amongst our study participants compared with their counterparts in developed countries who received more transfusions.

Secondly, many other studies have reported higher percentages of RBC alloimmunisation in haemoglobinopathies, such as SCD or thalassaemia, including Uganda^3^ (SCD, alloAb 6.1% amongst 428 patients), Tunisia^21^ (SCD and thalassaemia, alloAb 7.8% amongst 309 patients), Italy^23^ (thalassaemia, alloAb 5% amongst 1435 patients) and Brazil^24^ (SCD, alloAb 9.9% amongst 828 patients). These studies included a significantly higher number of patients; thus, the relatively low number of patients in our study might be a second limiting factor. The low rates in our study also differ from studies conducted in populations where there is high heterogeneity between donors and patients. In a study by Rosse et al.,^10^ involving 1814 SCD patients with an RBC alloimmunisation rate of 18.6%, the donors were of European-American ancestry and the SCD patients were of African-American ancestry.

Thirdly, patients in our study were predominantly children aged 16 years or younger (*n* = 159; 70%), 14% were aged ≤ 3 years. Studies of paediatric patients have reported lower RBC alloimmunisation rates. Aygun et al.^25^ and Sarnaik et al.^21^ concluded that children with SCD who were hypertransfused had a lower frequency of alloimmunisation as compared with adults.^21^ Another study involving 167 paediatric and 62 adult SCD patients supported this observation, where the rates of allo- and autoimmunisation in children and adults were 29% and 8%; 47% and 9.7%, respectively.^26^ Other authors advocate that transfusion started when patients are young (aged 1–3 years) may induce immune tolerance against alloimmunisation.^9^ The fact that 14% of our study patients were aged ≤ 3 years could have contributed to the low rate of RBC alloAb formation that we observed.

Fourth, the prevalence of RBC alloimmunisation amongst our cancer patients was low (2.2%), with only two HM patients and no alloAbs amongst SM patients. This is lower than that in the Ugandan study, where the rate was 8.3% amongst cancer patients. Shahida et al.^22^ studied 150 cancer patients who had at least five transfusions and found the prevalence rate of alloAbs to be 6%. In a study by Seyfried and Walewska^11^ of 1502 multi-transfused patients, the overall incidence of alloAbs was 5.7%, with the lowest rate found amongst patients with lymphoproliferative syndromes (1.8%).^11^ Of note, all the cancer patients in our study were undergoing chemotherapy at the time of transfusion. It has been observed that patients with progressive malignancies undergoing intensive chemotherapy tend to have a low antibody formation response to foreign antigens.^26,27,28^

Finally, a majority (57%) of our donor population expressed the Rh formula of ccD.ee, which could partly explain why we did not find RBC alloantibodies directed against highly immunogenic antigens such as D or E. A study by Badjie et al.,^29^ conducted amongst 800 donors from various ethnic groups, found the prevalence of the ccD.ee phenotype to be 81.9% in East Africa and a study by Baby et al.^30^ found a prevalence of 67.9% in West Africa (Mali). These results suggest that a large proportion of donors – exceeding 50% – and transfusion recipients in Africa share equal Rh phenotypes, so that Rh antibodies may be less frequently induced than in other parts of the world. This view is also supported by the low numbers for the ‘rr’ (Rhesus-D negative) phenotype amongst our donor group (only 4%). This phenomenon might be also true for Kell antibody formation, as we found no Kell positive individuals amongst our donors. It has been reported that more than 98% of black Africans are Kell negative.^30,31^

We found a positive DAT in 32 (14.0%) patients, with a subgroup of seven IgG warm autoAbs, which can induce significant clinical autoimmune haemolysis. We did not seek information about the presence of autoimmune haemolytic anaemia in these patients, because this can be clinically asymptomatic and the reaction can be masked by the severity of the underlying disease and lack of adequate post-transfusion records.

### Limitations

It has been reported that 25% of alloantibodies become undetectable within a median of 10 months of follow-up, which may lead to the underestimation of the prevalence of antibodies formed.^10,32^ This can result in a patient receiving RBCs and consequently experiencing a secondary immune response that may compromise the benefit of the following transfusion.^33^ Because this was a cross-sectional study, some RBC alloantibodies might have been missed, since they have been reported to disappear with time.^32,34^ Other factors that might also be responsible for the disparity in results include: the fact that the majority of the study patients were children; low mean of transfused units; inability to meet optimal transfusion needs for these patient groups; and the frequency of testing.

### Conclusion

In this study, we observed a low rate of RBC alloimmunisation amongst both SCD and cancer patients. The low numbers of transfusions and transfusion events that are currently being applied at KNH and the relatively homogeneous distribution of Rh-/K-RBC alloantigens amongst Kenyan donors provide an explanation for the low alloAb frequency amongst Kenyan transfusion recipients. At the current stage of the Kenya Health Care System, routine antibody screenings or extended RBC antigen matching do not seem to be justified, as the relatively homogenous RBC alloantigen distribution of Kenyan blood donors provides at least some protection from immune RBC alloAb formation. However, with improvements in health care, more SCD and haemato-oncology patients are likely to receive a more intensive transfusion treatment, which could lead to an increased risk of RBC alloimmunisation. Therefore, further development of the healthcare system in Kenya will require a thorough reconsideration of the pretransfusion laboratory practice, in particular, if transfusion frequencies increase and/or donor groups change.

## Trustworthiness

This study reflects the findings obtained from laboratory testing and analysis as observed by the technical group.

### Reliability and validity

The experimental design and procedures used in this study are reliable and valid as they have been used previously in other studies, most of which are cited in this article. The results of the experiments in this article were obtained using specimens collected in various clinics at Kenyatta National Hospital, Kenya and were analysed using standard procedures in Hannover, Germany.
